# Wnt signaling in multiple myeloma: a central player in disease with therapeutic potential

**DOI:** 10.1186/s13045-018-0615-3

**Published:** 2018-05-18

**Authors:** Ingrid Spaan, Reinier A. Raymakers, Anja van de Stolpe, Victor Peperzak

**Affiliations:** 1 Laboratory of Translational Immunology, University Medical Center Utrecht, Heidelberglaan 100, 3584 CX Utrecht, the Netherlands; 2 Department of Hematology, University Medical Center Utrecht, Heidelberglaan 100, 3584 CX Utrecht, the Netherlands; 3 Molecular Diagnostics, Philips Research, High Tech Campus 11, 5656 AE Eindhoven, the Netherlands

**Keywords:** Multiple myeloma, Wnt signaling, β-catenin, Osteolytic bone disease, Drug resistance, Targeted therapy

## Abstract

Multiple myeloma is the second most frequent hematological malignancy in the western world and remains incurable, predominantly due to acquired drug resistance and disease relapse. The highly conserved Wnt signal transduction pathway, which plays a key role in regulating cellular processes of proliferation, differentiation, migration, and stem cell self-renewal, is associated with multiple aspects of disease. Bone homeostasis is severely disturbed by Wnt antagonists that are secreted by the malignant plasma cells in the bone marrow. In the vast majority of patients, this results in osteolytic bone disease, which is associated with bone pain and pathological fractures and was reported to facilitate disease progression. More recently, cumulative evidence also indicates the importance of intrinsic Wnt signaling in the survival of multiple myeloma cells. However, Wnt pathway-activating gene mutations could not be identified. The search for factors or processes responsible for Wnt pathway activation currently focuses on aberrant ligand levels in the bone marrow microenvironment, increased expression of Wnt transcriptional co-factors and associated micro-RNAs, and disturbed epigenetics and post-translational modification processes. Furthermore, Wnt pathway activation is associated with acquired cell adhesion-mediated resistance of multiple myeloma cells to conventional drug therapies, including doxorubicin and lenalidomide. In this review, we present an overview of the relevance of Wnt signaling in multiple myeloma and highlight the Wnt pathway as a potential therapeutic target for this disease.

## Background

Multiple myeloma (MM) is a neoplastic disorder that is characterized by the infiltration and clonal proliferation of antibody-secreting post-germinal center plasma cells (PCs) in the bone marrow (BM). It is the second most frequent hematological malignancy, accounting for approximately 10% of all hematological cancers, and particularly affects the elderly with a median age at diagnosis of 69 years [1, 2]. MM is typically preceded by a pre-malignant state, which is referred to as monoclonal gammopathy of undetermined significance (MGUS). It is estimated that approximately 3–4% of the population above the age of 50 acquire this condition, but risk of progressing to MM is limited to 0.5–1% per year [3]. When detected monoclonal protein is accompanied by a clonal BM PC count exceeding 10%, the diagnosis MM is set [3]. Intramedullary MM may first present in an asymptomatic phase, often referred to as smoldering myeloma (SMM), but progression to active disease is significant within the first 5 years after onset [4]. Active disease is characterized by myeloma-related end organ failure and includes renal insufficiency, anemia, bone disease, and subsequent hypercalcemia as a result of the excess bone resorption [5]. Especially the presence of osteolytic bone disease, which develops in 80–90% of patients, is a major disease burden and often results in severe bone pain and detrimental pathological fractures [6]. Development of novel pharmaceutical agents has resulted in major advances in the treatment of MM in the last two decades [7]. In particular, co-treatment strategies of immune-modulatory drugs, proteasome inhibitors, conventional chemotherapy, and recently also monoclonal antibodies resulted in substantial progression in treatment outcome. However, despite the improvements in patient survival rates and quality of life, MM remains an incurable disease, with a current median survival of 6 to 7 years [2].

The Wnt signal transduction pathway is a highly evolutionary conserved and pleiotropic cell-to-cell communication route that regulates cellular processes including proliferation, differentiation, and fate determination. During embryogenesis, it functions as a concentration-dependent and long-range morphogen in establishing body axis formation [8]. In mature organisms, Wnt signaling is predominantly known for its role in the regulation of tissue-specific stem cell populations, thereby maintaining adult tissue homeostasis [9]. This has been confirmed in colon epithelium and skin tissue and has now also been reported in the hematopoietic stem cells (HSCs) that give rise to all blood cell lineages [10]. Deregulation of the Wnt pathway is associated with diverse human pathologies, cancer in particular. Although it was primarily recognized for its role in colorectal cancers, many more malignancies have been linked to aberrant Wnt pathway activation. Mutations in crucial Wnt signaling components, including APC, Axin, β-catenin, and Wnt ligands themselves have been reported in gastrointestinal cancers [11], pancreatic ductal adenocarcinoma [12], melanoma [13], lung cancer [14], cervical cancer [15], mammary carcinoma [16], prostate cancer [17], and tumors of the central nervous system [18] and result in hyperactivation of the Wnt pathway, ultimately increasing proliferation of malignant cells. Besides its reported role in the initiation and development of these solid tumors, there is now an increasing body of evidence that indicates involvement of Wnt signaling in hematological malignancies as well, including leukemias and MM [19].

The relevance of Wnt signaling in MM was first acknowledged for its regulating role in bone homeostasis. Wnt signaling tightly controls the balance between bone-forming osteoblasts and bone-resorbing osteoclasts, both by direct and indirect mechanisms [20]. Malignant PCs in MM severely disturb this system by secretion of Wnt antagonists in the BM microenvironment. This skews the balance toward osteogenic bone resorption and results in development of the characteristic osteolytic bone lesions [21]. In this process, a plethora of growth factors that were embedded in the bone matrix are released, which subsequently enhance MM cell growth and survival [22]. This coupled interaction essentially creates a vicious cycle of destructive bone disease and MM disease progression.

In recent years, more emphasis was put on the biological significance of active Wnt signaling intrinsic in MM malignant PCs. Human multiple myeloma cell lines (HMCLs) were found to express multiple Wnt receptors, co-receptors, and downstream pathway mediators that stabilized in response to ligand-mediated pathway activation [23]. Furthermore, HMCLs as well as primary PC isolates from MM patients overexpress the transcriptional co-activator β-catenin, including the active non-phosphorylated variant which is key to Wnt target gene transcription upon pathway activation [24]. The biological consequence of active Wnt signaling in these cells remains a point of discussion however. Multiple in vitro and in vivo studies used various approaches to either activate or inhibit Wnt signaling in MM cells and reported contradictory results on cell proliferation, morphology and disease progression. Also, the mechanistic role of pathway activation remains elusive, since no mutations in the genes of any of the major pathway mediators, including APC, Axin, and β-catenin itself, could be detected [24]. Current research focuses on aberrant levels of ligands in the BM microenvironment, increased expression of transcriptional co-factors, including BCL9 and associated micro-RNAs, as well as disturbed epigenetics and post-translational modification processes including sumoylation, as factors contributing to malignant Wnt signaling in MM.

In addition to canonical β-catenin-mediated transcriptional effects, Wnt signaling can also affect cell morphology, migration, and adhesion in a more direct way via the non-canonical Wnt/planar cell polarity (PCP) pathway. By acting on the small GTPases that regulate cytoskeleton formation, Wnt signaling can increase adhesion of malignant PCs to BM stromal cells and thereby enhance drug resistance [25]. The acquisition of resistance to conventional therapies in disease relapse is an emerging clinical problem. Targeting the Wnt pathway could therefore be an interesting new avenue in the treatment of MM.

Here, we present a complete overview of the relevance of Wnt signaling in MM. We summarize the role of Wnt signaling in MM bone disease, discuss the relevant studies performed in the context of intrinsic Wnt signaling in MM pathogenesis, both focusing on the biological significance as well as mechanisms responsible for signaling activation, and highlight the role of Wnt signaling in drug resistance and its potential as a therapeutic target in the treatment of MM.

## The WNT signaling pathway

The Wnt family currently consists of 19 secreted lipid-modified glycoproteins that can signal through one or more of the Wnt signal transduction pathways and can do so in a paracrine or autocrine fashion. They exhibit unique expression patterns and developmental functions, but were previously classified based on their ability to induce transformation of the epithelial cell line C57MG. Highly transforming members are thought to signal mainly through the β-catenin-dependent canonical Wnt pathway and include Wnt1, Wnt2, Wnt3, and Wnt3a, while the non-transforming members Wnt4, Wnt5a, Wnt5b, and Wnt7b were reported to have no effects on transformation and are likely to signal towards the non-canonical Wnt pathways. Several additional Wnts including Wnt6 and Wnt7a do not belong to these categories and are classified as intermediate transforming members, resulting in weak morphological changes [26]. There is only very little information about the structure of Wnts, since they are by nature difficult to synthesize in a recombinant setting, insoluble, and problematically complex to purify. Only in 2012, researchers succeeded in elucidating the X-ray crystal structure of *Xenopus laevis* XWnt8 [27]. Human Wnts are all very similar in size, between 39 and 46 kDa, and all contain 22 to 24 highly conserved cysteine residues that determine protein folding. All Wnt ligands go through an extensive process of post-translational modification before they become secreted. Both transforming and non-transforming members become glycosylated in the endoplasmic reticulum (ER); however, glycosylation of both Wnt1 and Wnt5a were reported to be indispensable for their functions [28]. In the ER, Wnts also become acylated. The membrane-bound O-acetyltransferase porcupine catalyzes the addition of palmitoleate groups to the conserved cysteine residues, which were found to be essential for progression of Wnts through the secretory pathway. Also, Wnt signaling capacity is diminished in absence of palmitoylation, most likely because these acyl groups mediate the interaction of the ligands with its receptors [29]. Additional post-translational modifications have been reported in very specific subgroups of Wnt ligands and include GPI anchorage to Wnt1 and Wnt3a and tyrosine sulfation of Wnt5a and Wnt11 [30]. Since Wnt proteins are so hydrophobic, they are mainly associated with the plasma membrane and extracellular matrix (ECM) [19]. Incorporation of Wnt ligands in membrane-enclosed vesicles, including exosomes, ensures adequate transport over larger distances of extracellular space [31].

**Fig. 1:**
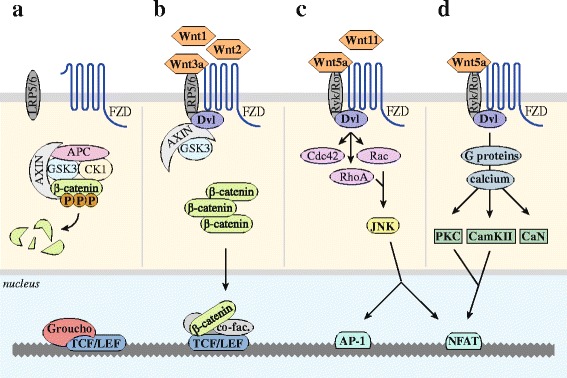
Schematic depiction of the canonical and non-canonical Wnt pathways. **a** When the canonical Wnt pathway is inactive, a cytosolic destruction complex consisting of Axin, APC, GSK3 and CK1 is formed. By phosphorylating β-catenin, this complex marks β-catenin for ubiquitination and subsequent degradation by the proteasome. The Wnt transcription factors TCF/LEF remain repressed by Groucho. **b** Upon binding of a canonical Wnt ligand to the Fzd receptor and LRP5/6 co-receptor, the canonical pathway becomes activated. Dvl is recruited to the receptor complex and subsequently recruits Axin-GSK3, thereby disrupting the destruction complex. Cytosolic levels of β-catenin stabilize and translocate to the nucleus. Here, β-catenin associates with the transcriptional machinery, transcriptional co-factors, and binds to TCF/LEF. This allows for active transcription of Wnt target genes. **c**, **d** Non-canonical Wnt ligands bind to Fzd and other transmembrane proteins such as Ryk and Ror, resulting in the recruitment of Dvl. **c** In the PCP pathway, this can result in activation of the small GTPases RhoA, Rac, and CDC42, which influence cytoskeleton function. RhoA and Rac can also activate JNK, and subsequent AP-1 and NFAT-mediated transcriptional programs. **d** In the Ca^2+^ pathway, Dvl activates heterotrimeric G proteins, resulting in a cytosolic calcium flux. This activates calcium-dependent signaling molecules including PKC, CamKII, and CaN. Both PKC and CamKII can also promote NFAT transcriptional activity

The canonical Wnt signaling pathway all revolves around the transcriptional co-activator β-catenin. When the Wnt pathway is inactive, continuously synthesized β-catenin is eliminated by a cytosolic destruction complex consisting of the scaffold proteins APC and Axin1 and the kinases GSK3 and CK1 (Fig. 1a). This destruction complex phosphorylates β-catenin at specific and highly conserved serine and threonine residues, thereby marking it for ubiquitination by the E3 ligase β-TrCP and subsequent proteasomal degradation [32]. Wnt signaling is activated upon binding of a Wnt ligand to its cognate receptor complex, consisting of the seven-span transmembrane protein frizzled (Fzd), of which ten isoforms are identified, and its co-receptors LRP5 and LRP6. Upon activation, the receptor complex recruits the effector protein disheveled (Dvl) to the plasma membrane, which is thought to result in subsequent recruitment of Axin1-GSK3, thereby disrupting the cytosolic destruction complex (Fig. 1b) [33]. Consequently, β-catenin is no longer phosphorylated and degraded but stabilized in the cytoplasm and able to translocate to the nucleus. Upon association with the basal transcriptional machinery and co-factors including pygopus and BCL9, β-catenin binds to members of the LEF/TCF family of transcription factors [34]. In this way, β-catenin facilitates transcription of Wnt target genes. These include cell cycle regulators like *CCND1* (encoding cyclinD1) and *MYC* and the survival molecule *BIRC5*, which translates into the inhibitor-of-apoptosis family member survivin. Survivin is overexpressed in a subset of MM patients and is correlated with multidrug resistance [35]. Also, *AXIN2*, a structural and functional homolog of Axin1, is transcribed upon β-catenin-LEF/TCF mediated transcription and thereby initiates a negative feedback loop upon canonical Wnt pathway activation [36].

In contrast to the canonical pathway, β-catenin-independent non-canonical Wnt pathways signal through the Fzd receptor without the need of the LRP co-receptor. Instead, association with other transmembrane proteins, including the receptor tyrosine kinases Ryk and Ror, has been reported. Multiple Wnt ligands including Wnt5a and Wnt11 are capable of doing so [37]. By recruiting Dvl these Wnts can activate multiple signaling cascades, which are vastly intertwined. In the Wnt/PCP pathway recruitment of Dvl results in activation of the small GTPases RhoA, Rac, and Cdc42, which actively rearrange the cell’s cytoskeleton and control cell polarity and motility (Fig. 1c) [38]. Additionally, RhoA and Rac are capable of activating Jun kinase (JNK), which can induce activation of AP1- and NFAT-mediated transcriptional programs [37]. In the Wnt/Ca^2+^ pathway, similar recruitment of Dvl results in activation of heterotrimeric G-proteins, which ultimately induce a calcium flux into the cytosol (Fig. 1d). Calcium-dependent signaling molecules such as PKC, camKII, and CaN subsequently become activated and influence a wide variety of signaling cascades, reliant on the cellular context [38]. Like JNK, both PKC and CamKII are capable of inducing NFAT-mediated transcription by facilitating its nuclear translocation [37].

The cellular outcome of active Wnt signaling is dictated by many factors, including the type(s) of Wnts present, as well as the receptor and co-receptor it engages with. Following the classification of Wnt ligands also Fzd receptors were divided into separate groups based on their ability to activate either canonical or non-canonical Wnt pathways upon overexpression [39]. However, from a biological standpoint these classification systems provide only limited information. The classical transforming Wnt3a is also capable of stabilizing β-catenin via non-canonical Rho [40], while the notorious non-canonical Wnt5a is able to activate the β-catenin-driven canonical Wnt pathway in the presence of Fzd5 in *Xenopus laevis* overexpression studies [41]. Many additional levels of regulation affect which downstream signaling cascades become activated. Varying concentrations of Wnt ligands in the microenvironment can induce differential target gene transcription. This is a direct consequence of the fact that Wnts create gradients to function as morphogens during embryonic development [9]. Expression of intracellular pathway mediators, including basal levels of β-catenin and differentially expressed isoforms of the LEF/TCF transcription factors, can also influence Wnt signaling and can even result in distinct cellular outcomes in identical Wnt ligand and receptor conditions [42]. Furthermore, the Wnt pathway can be associated with, and influenced by, other cell signaling pathways, including the PI3K/Akt, FGF, Notch, and Hedgehog signaling pathways [43]. In addition, a growing list of Wnt antagonists is currently being identified. Extracellular inhibitors comprise soluble secreted Fzd-related proteins (sFRP)1-5 that act as decoy receptors by directly binding to extracellular Wnts, resulting in a concentration-dependent downregulation of general Wnt pathway activation. This in contrast to Dkk1-4, which specifically antagonizes canonical Wnt signaling by binding to extracellular subregions of the LRP co-receptors. Other extracellular antagonists are Wnt inhibitory factor 1 (WIF1) and the bone-specific Wnt inhibitor SOST/sclerostin [44]. The best characterized intracellular Wnt pathway inhibitor is ICAT, which inhibits the interaction between β-catenin and the transcriptional complex members LEF/TCF and p300 [45]. All these different levels of control combined ensure tight regulation of the potent Wnt signal transduction pathway.

## WNT signaling in multiple myeloma bone disease

MM cells preferentially home to the BM as their host organ, which is a very active site of Wnt signaling. The BM is a soft, well vascularized and highly spatially organized tissue that harbors a diverse cellular content consisting of bone-forming osteoblasts and bone-resorbing osteoclasts, BM adipocytes, endothelial cells, BM stromal cells, mesenchymal stem cells (MSC), and HSC [46]. The latter two stem cell populations reside in specific BM niches, where their stemness potential is tightly regulated by distinct niche signaling pathways that allegedly includes canonical Wnt signaling [47]. Furthermore, the BM hosts a wide variety of innate and adaptive immune cells including macrophages, neutrophils, and diverse B and T cell populations. All these cells are joint by an extensive network of ECM proteins and fluid, which contain a variety of growth factors and cytokines [48]. Many of the cells present in the BM produce a range of Wnts, both of the canonical and non-canonical branches. MSC do not only rely on intrinsic Wnt signaling to maintain their stem cell features, they also secrete Wnt ligands, including Wnt2, Wnt4, Wnt5a, Wnt11, and Wnt16 [49]. Additional major sources of Wnts are endothelial cells and BM stromal cells. Several immune cells were also shown to be able to contribute to Wnt signaling, including monocytes, dendritic cells, and macrophages [50]. When located in the BM, T cells also express the canonical ligand Wnt10b, which is positively associated with bone formation [51].Additionally, cells of the osteoblast and osteoclast lineages themselves secrete Wnt5 and Wnt10b that function as part of their intercellular communication pathways [52, 53].

### Wnt signaling is a master regulator of bone homeostasis

MM cells interrupt Wnt signaling in the BM and thereby severely disturb bone homeostasis. Essentially, bone metabolism is controlled by three cell types: osteoblasts, osteocytes, and osteoclasts. Osteoblasts are derived from pluripotent MSCs, which also produce fibroblasts, chondrocytes, myoblasts, and adipocytes. Wnt signaling has a major impact on this differentiation process by directing MSC differentiation away from the chrondrocytic and adipocytic lineages, towards differentiation into osteoblasts [54]. Additionally, Wnt signaling was proven to promote survival of these osteoblasts, at least partly via signaling through the Src/ERK and PI3K/Akt pathways [55]. Mature osteoblasts reside in specific niches that are separated from the rest of the BM by a single layer of bone-lining canopy cells [56]. The osteoblasts are responsible for the formation of new bone tissue during the continuous bone modeling and remodeling processes and do so by secreting bone matrix components, including collagen, and regulating mineralization of the bone tissue [56]. During this process they can become embedded in the bone tissue, which promotes their terminal differentiation into osteocytes.

Osteocytes comprise approximately 95% of all cells present in the bone matrix and are the only cell type identified in bone that possess a significantly active intrinsic canonical Wnt signaling pathway [57]. Within the mineralized matrix, they influence bone formation by secreting the Wnt antagonist SOST/sclerostin [58]. These factors diffuse from the osteocyte lacuna, via a canalicular network, to the bone surface, where they inhibit osteoblastic activity [57]. The main function of osteocytes is to regulate bone mass in the context of mechanical loading. As a result of the mechano-transduction pathway, significant mechanical stress directly leads to a downregulation of Wnt inhibitor secretion levels and a subsequent gain in bone mass to maintain skeletal integrity [59].

Osteoclasts are the natural counterpart of osteoblasts and facilitate the process of bone remodeling by resorbing bone tissue. They do so by secreting lytic enzymes that degrade the bone matrix [60]. Osteoclasts arise from monocyte/macrophage hematopoietic precursor cells that reach the BM via the vasculature [56]. Fusion of multiple of these cells results in the characteristic multinucleated cells. However, for osteoclasts to become fully differentiated, several osteoblast-secreted factors including RANKL are required [61]. Like osteoblasts, osteoclasts and their precursors are influenced by Wnt signaling. However, the biological consequences of Wnt signaling are rather complex, since Wnt ligands were found to stimulate early steps of osteoclastogenesis, while inhibiting the final differentiation stages [62].

**Fig. 2:**
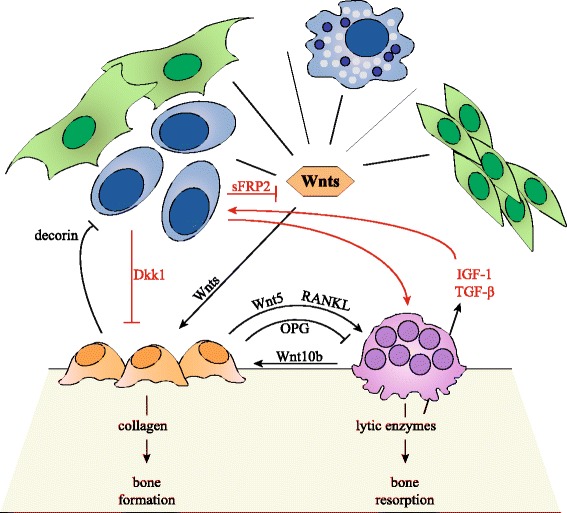
Bone homeostasis and multiple myeloma-induced osteolytic bone disease. (In black) In the BM, Wnt ligands are expressed by MSCs, endothelial cells, BM stromal cells, immune cells including macrophages and dendritic cells, osteoblasts and osteoclast. Wnts promote differentiation of MSCs towards the osteoblastic lineage, stimulate osteoblast proliferation, and promote osteoblast survival. Osteoblasts regulate osteoclast activity by secreting the antagonistic molecules RANKL and OPG, and Wnt5. Osteoclasts also regulate osteoblast function by secreting Wnt10b. Through this system, osteoblast and osteoclast activity is coupled and bone homeostasis is maintained. Osteoblasts also inhibit MM cell growth by expressing decorin. (In red) MM cells promote osteoclast function by secreting RANKL. Osteoclasts stimulate MM cells by secreting IL-6 and Annexin II. Enhanced bone resorption also leads to increased IGF-1 and TGF-β levels. MM cells also inhibit osteoblasts by secreting Wnt inhibitors sFRP2 and Dkk1. This disrupts the osteoblast-osteoclast balance and bone homeostasis and leads to osteolytic bone disease

In healthy human adults, bone remodeling is executed to replenish old bone, strengthen bone tissue in response to physical stress, or at sites of injury. Osteoclasts resorb the compromised bone tissue, after which adjacent cells, and presumably also tissue specific macrophages called osteomacs, clean the affected surface. The osteoclasts then recruit and stimulate osteoblasts to form new bone tissue in order to fill the created space. To balance bone resorption with equal bone formation, osteoblast and osteoclast activity is matched, which is referred to as coupling (Fig. 2, black arrows) [56]. The Wnt signaling pathway is found to be directly involved in this coupling mechanism. Bone-forming osteoblasts secrete non-canonical Wnt5a, which is a ligand for the Fzd-Ror2 and Fzd-Ryk receptor complexes that are present on the cell surface of osteoclast precursors. Subsequent activation of the downstream signaling cascade stimulates differentiation into more mature bone-resorbing osteoclasts [52, 63]. These osteoclasts in turn express multiple factors, including chemoattractants that recruit osteoblast precursors to the site of bone remodeling, and Wnt10b that stimulates bone formation, creating an ingenious feedback/feedforward loop of bone remodeling [53]. Besides these direct effects, Wnt signaling also plays a more indirect role in bone remodeling by influencing the RANK/RANKL/OPG signaling axis; one of the best characterized coupling routes [64]. The receptor RANK, which is present on the plasma membrane of osteoclasts, can be activated by binding of its cognate ligand RANKL that is predominantly expressed by osteoblasts. In addition, osteoblasts are also capable of secreting the natural decoy receptor OPG, which competes with RANK for the binding of RANKL. The balance between RANKL and OPG determines the level of signal transduction and thereby regulates osteoclast activation [65]. Both RANKL and OPG were shown to be direct Wnt target genes in osteoblasts. Increased Wnt signaling is associated with high secretion levels of OPG, but negatively regulates RANKL expression, which subsequently results in impaired osteoclastogenesis [66, 67].

### Multiple myeloma cells disrupt bone homeostasis by interfering with Wnt signaling

Like their benign counterparts, malignant PCs in MM express high levels of CXCR4 on their cell surface in order to home the BM [68]. Here, they adhere to BM stromal cells that create a permissive environment by expressing adhesion molecules and secreting an array of growth factors, chemokines, and other molecules [69]. By this direct cell-to-cell contact, MM cells also instruct BM stromal cells to produce high levels of osteoclastogenic stimuli, including RANKL, to augment bone resorption [70]. More importantly, BM infiltration allows MM cells to exert their effects on osteoblasts and osteoclasts in a more direct fashion, which ultimately facilitates tumor progression (Fig. 2, red arrows).

MM bone disease is characterized by both an increase in osteoclast activity, as well as a decrease in the number of osteoblasts. Osteoclastogenesis is stimulated in a direct fashion by the expression and secretion of osteoclast-activating factors such as RANKL by MM cells themselves, resulting in increased bone resorption [71]. As a consequence of bone resorption, a plethora of immobilized growth factors, including insulin-like growth factor and TGF-β [6], as well as calcium and ECM proteins, are released from the bone matrix. All these factors contribute to MM cell growth and survival [48]. Subsequent disease progression then results in an additional gain in bone tissue lysis, giving rise to a vicious cycle. In addition, osteoclasts themselves also secrete multiple factors to sustain MM cells, including the growth factor IL6 and survival factors BAFF and APRIL, and are believed to promote angiogenesis by secreting the pro-angiogenic factor osteopontin [72, 73]. Besides increased osteoclastogenesis, MM cells also reduce the number and activity of osteoblasts [64]. This not only enhances osteolytic bone disease by preventing new bone formation; osteoblasts were found to reduce MM cell growth, i.e., by producing a proteoglycan called decorin that was reported to induce apoptosis of MM cells [74].

Presumably, the most important mechanism by which MM cells promote an osteoblast/osteoclast disbalance is by attenuating active canonical Wnt signaling. MM cells, but not PCs isolated from MGUS patients, were found to express high levels of the canonical Wnt inhibitor Dkk1. Mouse model studies had previously shown that Dkk1 plays a crucial role in bone homeostasis. Overexpression of Dkk1 resulted in a decrease in bone mass, leading to osteopenia, while increased bone formation and bone mass was observed in single allele Dkk1 knockout mice [75]. The increased level of Dkk1 present in serum of MM patients was found to correlate with the presence and extend of bone lesions [76]. In vitro, serum of MM patients was shown to inhibit osteoblast differentiation, which could be restored by the addition of a Dkk1 neutralizing antibody [76]. In a more recent study, Yaccoby et al. used the murine SCID-rab model, in which mouse BM is substituted by rabbit BM through subcutaneous implantation of rabbit bone, to study the effects of Dkk1. Treatment with an anti-Dkk1 antibody, after engraftment of Dkk1 expressing primary MM cells, resulted in a reduced number of osteoclasts, enhanced number of osteoblasts, decreased bone resorption, and a subsequent decrease in tumor burden [77]. Edwards et al. reported similar results when C57BL/KaLwRij mice were intravenously inoculated with murine 5TGM1 MM cells, after which Wnt signaling was activated by treatment with the potent GSK3 inhibitor lithium chloride (LiCl). Although tumor burden in the BM was reduced, increased tumor growth was observed when the 5TGM1 cells were engrafted subcutaneously [78]. Qiang et al. showed that SCID-hu mice, which are comparable to the SCID-rab model but transplanted with human fetal bone, could be engrafted with the HMCL NCI-H929, which was stably transfected with either Wnt3a or empty vector control. Also in this model, increased osteoblast/osteoclast ratios were reported, accompanied by a reduction in tumor burden. In addition, recombinant Wnt3a treatment of SCID-hu mice carrying primary MM cells resulted in attenuation of bone resorption and MM cell growth [79]. These findings combined have culminated in clinical testing of the human neutralizing anti-Dkk1 monoclonal antibody BHQ880. Results from the phase 1b study, in which 28 patients with relapsed or refractory MM were enrolled, showed a general trend towards increased bone mass over time, particularly in the spine [80]. In 2013, a phase 2 study in SMM patients with high risk of disease progression was completed and also in this study, evidence of anabolic bone activity in a subset of patients was reported [81].

Although the effects of Dkk1 secretion on MM pathogenesis are most extensively characterized, MM cells have additionally been proven to express the Wnt antagonists sFRP2, sFRP3, and SOST/sclerostin. Serum levels of these Wnt inhibitors also correlate with the extend of MM bone disease [46]. Interestingly, in vitro treatment of osteoblasts with sFRP2 resulted in impaired differentiation capacity of these cells, similar to what was observed for Dkk1 [82]. Additional studies are required to elucidate the exact effects of these antagonists on MM pathogenesis.

## Intrinsic WNT signaling in multiple myeloma cells

In recent years, several studies were published indicating that MM cells do not only disturb Wnt signaling in the BM by secreting Wnt antagonists but also have the potential of an active intrinsic canonical Wnt signaling pathway. This was first evidenced by Qiang et al., who reported that HMCLs can express up to nine isoforms of the Fzd receptor that are co-expressed with the canonical co-receptor LRP6 and in most instances also LRP5 [23]. Although this was only studied on the mRNA level, treatment of these cell lines with Wnt ligand was found to stabilize transcriptionally active β-catenin, suggesting that these receptors were also functionally active [23]. In a similar study, the same HMCLs were found to also produce a range of Wnt ligands [83]. The presence of Fzd1, Fzd6, Fzd7, Wnt5A, and Wnt11 was later confirmed in a panel of primary PC samples isolated from MM patients and suggests that MM cells can stimulate their Wnt signaling pathway in an autocrine fashion [84]. However, since this analysis was also performed on the mRNA level, it is difficult to estimate how the subsequent protein Wnt ligand concentrations would relate to Wnts produced by other cells in the BM.

The presence of an active intrinsic Wnt signaling pathway in MM cells was proven to be quite specific for malignant PCs. According to current hypotheses, the canonical Wnt signaling pathway is active in B cells during early stages of development, but is terminated upon expression of IgM by immature B cells [85]. In accordance with this model, several malignant mature B cell lines only expressed high levels of Fzd3 and did not show any expression of LRP5 or LRP6 [23]. This suggests that the final maturation step of activated mature B cells towards PCs, which is accompanied by extensive genetic rearrangements, could be essential for the cells to be able to reactivate the canonical Wnt signaling pathway during malignant transformation.

### Wnt signaling is indispensable for normal B cell development

B cells, like all cells from the blood lineage, arise from multipotent HSCs that have the ability of self-renewal, as well as the ability to differentiate into more committed precursor cells through a process referred to as asymmetric cell division. Optimal regulation of these processes is required in order to maintain tissue homeostasis and to prevent depletion of the stem cell pool. Therefore, HSCs reside in specific BM stem cell niches, where their fate is tightly regulated by several distinct microenvironmental cell signaling pathways [85]. The exact role of Wnt signaling in this system is still under debate. The presence of an active canonical Wnt pathway in HSC in vivo was first confirmed by Reya et al. in 2003 [86]. However, several subsequent studies reported opposite results on the effect of Wnt signaling on the processes of HSC self-renewal and the capacity to reconstitute upon transplantation [87, 88]. Recent evidence now suggests that these contradictory observations result from differences in signaling levels. As Wnts function as morphogens and are normally present in concentration gradients within tissues, varying signaling levels can have differential cellular outcomes [89]. By introducing several distinct mutations in the APC gene in a conditional mouse model, Luis et al. were able to create a gradient of five different Wnt signaling levels. The results showed that a mild twofold increase in Wnt signaling level enhances HSC function through a sixfold increase in repopulation capacity upon transplantation. This is in contrast with intermediate or higher Wnt signaling levels, which completely impaired HSC self-renewal potential [89]. This data, in combination with a previously published study that revealed that a nearly complete blockage of the Wnt pathway in Wnt3a-deficient mice is detrimental for HSC function, highlights the complexity of Wnt signaling in this system [90].

**Fig. 3:**
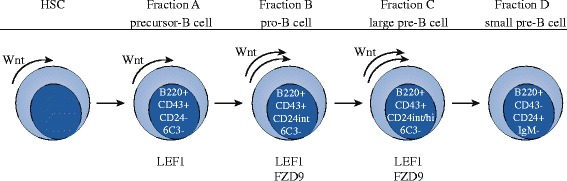
Wnt signaling in hematopoietic stem cells and early B cell development. Wnt signaling is essential for HSC self-renewal capacity and is also active in precursor cells from fraction A. Fraction B pro-B cells, that undergo IgH rearrangement, have an active Wnt pathway and express high levels of LEF1 and Fzd9. Also, large pre-B cells from fraction C that greatly expand their population express LEF1 and Fzd9 and have an active Wnt pathway. Small pre-B cells from fraction D stop proliferating and start rearranging IgL genes. Upon expression of IgM, Wnt pathway activity is expected to be terminated

In contrast to HSC biology, the role of canonical Wnt signaling in subsequent early stages of B cell development was more robustly established (Fig. 3). Already in 2000, Reya et al. reported that expression of LEF1 is confined to precursor B cells, pro-B cells, and IgM-negative pre-B cells [91]. Canonical Wnt signaling at these stages is thought to solely occur through the LEF1 isoform of the TCF/LEF family, since TCF members are the commitment factor for T cell development and are therefore expected to be specifically downregulated during B cell development [92]. Analysis of LEF1 knockout mice revealed an up to eightfold decrease in the number of pre-B and pro-B cells, accompanied by an increase in apoptotic cell numbers, which could be partly rescued by treatment with the GSK3 inhibitor LiCl. In addition, in vitro stimulation of isolated LEF1 knockout pro-B cells with Wnt3a-conditioned medium only resulted in stabilization of free β-catenin, while this was accompanied by cell cycle activation and proliferation in wild-type pro-B cells [91]. Similar findings were reported by Ranheim et al., who utilized a Fzd9 knockout mouse model to study the effects of canonical Wnt signaling on B cell development. Fzd9 was found to be exclusively expressed in pro-B cells and large pre-B cells. Knockdown of Fzd9 resulted in a depletion of developing B cells, which was most pronounced in the large pre-B cell population [93].

Although both abovementioned studies suggest that canonical Wnt signaling is terminated when the final major round of pre-B cell division is completed, Yu et al. found evidence that canonical Wnt signaling is also required for normal PC function. When β-catenin was specifically deleted in the B cell lineage in a conditional mouse model, a small but significant reduction in PC generation was observed upon stimulation with bacterial LPS. Further analysis showed that the reduction in PC formation was accompanied by decreased expression levels of the transcription factors Blimp-1 and IRF-4, which are both essential for PC differentiation [94].

### Activation of Wnt signaling in multiple myeloma cells is facilitated by multiple processes

With previous studies taken into account, one could state that canonical Wnt signaling is of importance during many of the major proliferative stages in the development and activation of B cells: HSC self-renewal, population expansion of developing B lymphocytes, and PC generation upon antigenic stimulation of mature B cells. From this perspective, (re)activation of the canonical Wnt pathway in MM cells, which evidently have an active cell cycle, seems intuitive. In addition, aberrant Wnt pathway activation is very often implicated in cancer. This accounts for solid tumors, as well as different types of leukemia [10, 19]. Indeed, Derksen et al. showed that both HMCLs and primary PC isolates from MM patients overexpress β-catenin, including the transcriptionally active subset. This is in contrast to mature B cells, memory B cells, and healthy PC populations [24]. However, it currently remains unclear what drives activation of the intrinsic Wnt/β-catenin pathway in MM cells. Extensive sequencing experiments did not reveal any detectable mutations in the major Wnt pathway mediators, including β-catenin itself (encoded by *CTNNB1*), *AXIN1*, or the tumor suppressor *APC*, as is normally observed in canonical Wnt-driven tumors like colon cancer [8].

First studies indicated a role for BCL9 in the activation of intrinsic Wnt signaling in MM. The *BCL9* gene, which is located on the frequently amplified 1q chromosomal region, was first identified in B cell acute lymphoblastic leukemia as a result of the t(1;14) translocation [95]. The nuclear protein was shown to be an important co-factor for transmission of downstream Wnt signaling by facilitating effective β-catenin-LEF/TCF cooperation [96]. Mani et al. reported that BCL9 transcript levels were specifically enhanced in a subset of HMCLs and primary PC samples from MM patients, compared to normal PCs. This was accompanied by a perinuclear localization of β-catenin, enhanced transcriptional activation, and an increase in cell proliferation, migration, and invasion. Furthermore, murine xenograft models transplanted with the HMCL MM1.s in which BCL9 was knocked down by shRNA, showed reduced tumor burden, decreased metastasis, and prolonged survival compared to shRNA control mice [97]. A follow-up study in 2014 identified the oncogenic BCL9 as a direct target of the tumor suppressor microRNA miR-30-5p. Levels of miR-30s are downregulated in a large subset of MM PC samples, which is thought to result from adhesive interactions of MM cells to BM stromal cells, and is inversely correlated with BCL9 expression. Ectopic expression of miR-30c in malignant PCs was associated with reduced levels of BCL9 mRNA and BCL9 protein and decreased Wnt target gene transcription, as was assessed by functional promoter-reporter assay and CD44 and Axin2 mRNA levels. Furthermore, HMCLs NCI-H929 and OPM1 overexpressing miR-30c showed a clear reduction in cell proliferation, migration, and invasion, accompanied by a mild increase in the number of apoptotic cells. In addition, miR-30c, as well as a miR-30mix, could resensitize the NCI-H929 cells to dexamethasone treatment, thereby revealing great therapeutic potential. These results were validated in vivo by the use of murine xenograft models [98].

Several studies also suggested a role for dysregulated post-translational modification of Wnt pathway mediators in aberrant intrinsic Wnt pathway activation in MM cells. In 2015, Huang et al. reported that β-catenin is subject to sumoylation [99]. Previous studies already indicated that SUMO protein expression levels are upregulated in carcinogenic cellular processes, including oxidative stress and apoptosis [100]. Furthermore, sumoylation is known to be aberrantly activated in a large subset of MM patients and hyperactive sumoylation is correlated with adverse patient prognosis [101]. By siRNA-mediated downregulation of the effector protein SUMO-1, Huang et al. were able to interfere with the sumoylation process in MM cells in vitro. This resulted in a proteasome-mediated downregulation of β-catenin protein levels, which was accompanied by a reduction in canonical Wnt signaling and decreased expression of Wnt target genes. Combined, this results in increased numbers of apoptotic cells and subsequent growth inhibition of HMCLs [99].

Aberrant epigenetics were suggested to be responsible for intrinsic Wnt pathway activation in MM cells by Chim et al., who analyzed DNA methylation of CpG islands, indicative of transcriptional repression and subsequent gene silencing, in the promoter regions of the Wnt antagonists WIF1, Dkk3, APC and sFRP1, sFRP2, sFRP4, and sFRP5. Of the 50 MM patients enrolled in this study, 42% showed abnormal methylation of at least one of these genes. Within this group, more than two thirds of the patients even showed methylation of two or more inhibitors. This is in contrast to healthy PC control samples, in which no methylation of any of these genes was detected [102].

Very recently, van Andel et al. proposed two additional mechanisms for Wnt/β-catenin activation in MM cells. The first hypothesis concerns the loss of the tumor suppressor deubiquitinating enzyme CYLD, which is a known negative regulator of NFκB, TGFβ, and Notch signaling [103–105]. A previous study by Tauriello et al. already reported that loss of CYLD results in hyperubiquitination of Dvl, thereby augmenting active complex formation at the plasma membrane upon Wnt stimulation and enhanced downstream β-catenin signaling levels [106]. The current study reports highly variable CYLD levels in both HMCLs and primary PC isolates from MM patients and correlates loss of CYLD, either by mutation or complete deletion of the locus, to MGUS progression and poor clinical prognosis in MM patients. Loss of CYLD in HMCLs was found to enhance β-catenin stabilization and localization to the nucleus, increase β-catenin-LEF/TCF reporter activity, and enhance MM cell growth and survival. Overall, loss of CYLD was associated with a Wnt-signaling gene expression signature [107]. Another study published by van Andel et al. in 2017 put more focus on the role of the BM microenvironment as a supportive niche for MM development. Numerous previous studies already showed the presence of R-spondins in the BM microenvironment, which are essential factors for osteoblastogenesis and thereby play an important role in bone development and homeostasis [108]. R-spondins are Wnt/β-catenin agonists and function by binding to their cognate receptors LGR4, LGR5, and LGR6 at the plasma membrane and subsequently regulate Wnt (co)-receptor turnover dynamics via the E3 ubiquitin ligases Rnf43 and Znrf3 [109]. The current study revealed aberrant expression of LGR4 in the majority of HMCLs, as well as primary PC isolates from MM patients. Interestingly, LGR4 overexpression is at least partially regulated via STAT-3 signaling, which in turn is mediated by IL-6, another cytokine that is vastly present in the BM microenvironment of MM patients. Simultaneous stimulation of LGR4-positive HMCLs with Wnt ligand and R-spondin resulted in a major increase in LRP6 phosphorylation, β-catenin stabilization and translocation to the nucleus, activation of a Wnt reporter construct and an increase in MM cell proliferation, while the LGR4-negative HMCL L363 did not show this. Stimulation with R-spondin alone did not result in Wnt pathway activation in the majority of cell lines [110]. This underlines the fact that aberrant R-spondin-LGR signaling only causes Wnt hypersensitivity in the context of an already active Wnt pathway, and not as a primary pathway activation event. In a very recent follow-up study Ren et al. indicated an important role for the heparan sulfate proteoglycan syndecan-1 in MM Wnt signaling activation. Syndecan-1 is an ECM-related transmembrane protein that is expressed by malignant PCs and to a lesser extent healthy PCs as well. It has previously been shown to be important for the connection between PCs and the BM microenvironment and furthermore impacts MM cell growth and survival [111]. In this study, Ren et al. showed that the heparan sulfate chains that decorate syndecan-1 are able to bind Wnt ligands, as well as R-spondin, and thereby facilitates active and efficient Wnt-receptor complex formation on the MM plasma membrane. Knockdown of the enzyme responsible for functional syndecan-1 assembly was found to result in a reduction of Wnt signaling and impaired cell growth of a panel of HMCLs [112].

### Intrinsic canonical Wnt signaling is crucial for multiple myeloma cell survival

Besides the controversial mechanism that is responsible for activation of the canonical Wnt pathway in MM cells, there are some additional observations that remain unclear. Although Derksen et al. showed that MM PCs have an aberrantly active Wnt pathway due to β-catenin overexpression, signal transduction in response to Wnt ligands remained functional. Additional stimulation by Wnt3a-conditioned medium, purified Wnt3a, treatment with LiCl, or ectopic expression of constitutively active β-catenin, all resulted in a further two - to fourfold increase in proliferation of HMCLs [24]. MM cells thus still respond to Wnt ligands, irrespective of an already constitutive active pathway. However, it is not clear if stimulation occurs in a paracrine fashion, as a result of microenvironment-derived Wnts, or in an autocrine fashion by the canonical ligands that are expressed by MM cells themselves, or combinations thereof.

The major topic for discussion, however, concerns the biological consequences of intrinsic canonical Wnt signaling in MM. In 2003, Qiang et al. reported that Wnt pathway stimulation of HMCLs by Wnt3a-conditioned medium resulted in activation of the canonical Wnt pathway. This was demonstrated by increased levels and activation of Dvl2 and Dvl3, cellular stabilization of transcriptionally active β-catenin and an approximate sixfold increase in Wnt activity using aTopFlash reporter assay that is indicative of TCF/LEF-mediated transcription. However, they did not report an effect on the level of cell proliferation. Instead, major morphological changes were observed: the MM cells acquired a fibroblast-like phenotype, started to become adherent and developed filopodia-like protrusions [23]. This was in strong contrast to what was published by Derksen et al. in 2004. Here, stimulation of the Wnt pathway by purified Wnt3a, subsequent stabilization of transcriptionally active β-catenin and increased TopFlash reporter activity was accompanied by a two to fourfold increase in cell proliferation. In this study, no morphological changes of MM cells were reported [24]. Interestingly, both authors included NCI-H929 in their panel of HMCLs, so these contradictory results cannot be explained by a differential use of cell lines. However, it is known that the exact cellular outcome of Wnt signaling is influenced by many additional factors, including the level of Wnt ligands present like was observed for HSC self-renewal capacity. Especially in the case of Wnt-conditioned medium, the actual concentration of Wnt ligand is difficult to determine. In 2007, in accordance with the last study, Sukhdeo et al. reported that termination of Wnt/β-catenin signaling inhibited MM cell proliferation. By treating HMCLs with the small molecular compound PKF115-584, which interferes with the transcriptionally active β-catenin-TCF/LEF complex, cell cycle arrest in the G1 phase was induced. This resulted in increased levels of cleaved caspases 3, 8, and 9 and a subsequent gain in the number of apoptotic cells [84].

Since all previous observations are based on in vitro experiments, several independent research groups used genetically engineered mouse models to gain more insight into the in vivo mechanism of action. Both Yaccoby et al. and Qiang et al. reported a reduction in MM tumor growth upon forced activation of the Wnt signaling pathway. Yaccoby et al. used the SCID-rab model and, upon transplantation with primary MM cells, treated these mice with antibodies to neutralize the canonical Wnt inhibitor Dkk1. This resulted in a general reduction of MM tumor burden [77]. In a slightly different approach, Qiang et al. stably transduced the HMCL NCI-H929 with a Wnt3a expression construct. Although subsequent stabilization of β-catenin was confirmed, this again did not result in increased cell growth in vitro or in vivo upon subcutaneous transplantation into SCID mice [79]. Edwards et al. used the C57BL/KaLwRij model, which was engrafted with murine 5TGM1 MM cell line, followed by treatment with the GSK3 inhibitor LiCl to stimulate canonical Wnt signaling. This resulted in a decrease in tumor burden in bone, but concurrently resulted in increased tumor growth when MM cells were inoculated subcutaneously. Furthermore, expression of dominant negative TCF4 did not affect the LiCl-induced reduction in tumor burden in bone [78]. This suggests that the reported observations might result from interference with molecular processes distinct from canonical Wnt signaling, which seems plausible since GSK3 is involved in many additional cellular processes. More recently, two similar studies that address Wnt signaling in MM mouse models were published by Dutta-Simmons et al. and Ashihara et al., that used a rather different approach. Instead of enforcing active Wnt signaling, the canonical pathway was inhibited by specific knockdown of β-catenin. The first study used a shRNA-mediated approach for efficient knockdown of β-catenin. After the in vitro observation that treatment with Wnt3a-conditioned medium enhanced cell proliferation in HMCLs, β-catenin knockdown was found to greatly reduce this effect. When these MM cells were intravenously injected into NOD/SCID mice, the β-catenin knockdown group showed a mean survival of 94 days, versus 25 days for control mice that were treated with a scrambled shRNA. In addition, the experimental group showed a significant reduction in tumor burden with less tumor nodules in the BM, as well as a dramatic decrease in the occurrence of metastasis compared to control mice [113]. A similar trend was reported by Ashihara et al., who subcutaneously inoculated BALB/c albino nude mice with HMCLs. After 3-4 weeks, mice were injected around the site of active tumor development with either β-catenin siRNA or a control scrambled siRNA. Efficient β-catenin knockdown on the protein level was found to be correlated with significantly reduced MM tumor volume. This was accompanied by decreased expression levels of the direct β-catenin target c-Myc and increased levels of cleaved caspase 3 [114].

Although the additional use of in vivo mouse models could not elucidate the exact molecular mechanism of Wnt signaling or its relevance for tumor growth, these studies, when combined with in vitro data, did reveal an interesting trend. Extra stimulation of the Wnt pathway did not confer a growth advantage for MM per se. However, almost complete termination of canonical Wnt signaling, either by knockdown of β-catenin or disruption of the transcriptionally active β-catenin-TCF/LEF complex, induced MM cell apoptosis and significantly reduced tumor growth. This is similar to what was observed for Wnt signaling in HSC biology and could be the direct result of essential growth factor deprivation.

The ultimate purpose of the canonical Wnt/β-catenin pathway is to activate transcription of Wnt target genes. In vitro*,* this process is studied by the TopFlash reporter assay, in which the cells are transfected with a promoter-reporter construct that contains multiple Wnt responsive elements (WRE) followed by the sequence encoding the luciferase enzyme. After addition of substrate, the amount of measurable product formed by the enzyme reflects the level of Wnt/β-catenin transcriptional activity. Some studies additionally analyze expression levels of up to three Wnt target genes, often including *MYC*, *CD44* and *CCND1*. Although these are all direct Wnt target genes, MYC and CD44 are both a-specific, since their activity is incorporated in and influenced by many other cell signaling pathways [115, 116]. Additionally, in MM *CCND1* is not an ideal candidate, since upregulation of this gene is very often observed, either due to gene duplication or chromosomal translocations involving the cyclin-D locus [117]. When Wnt target gene activation upon stimulation of MM cells with Wnt3a was studied on a larger scale by microarray, no significant increase in the expression level of the Wnt target gene panel was observed [64]. One explanation for this observation is that Wnt/β-catenin-mediated transcription in MM cells is thought to occur solely through LEF1 of the TCF/LEF family. The *LEF1* gene is however quite distinct from the TCF genes. Whereas all TCFs are able to produce an “E” tail isoform at their carboxyl terminus, the *LEF1* gene does not encode such a tail at all. This “E” tail, which encodes a DNA-binding domain and a region that facilitates interaction with p300, was shown to be essential for the regulation of multiple Wnt target genes. The TopFlash reporter, with the multimerized WREs, is however so optimally constructed that reporter activation is tail-independent [118]. This could at least partially explain why a positive TopFlash assay is not necessarily accompanied by robust Wnt target gene expression upon Wnt stimulation. However, Sukhdeo et al. showed that several HMCLs additionally express TCF-4, further complicating the matter [84]. Another explanation could be that almost all Wnt target genes are identified from studies performed in colon cancer. The intestine, as well as the skin, is a site of very active canonical Wnt signaling by nature. These levels are more modest in mammary tissue and the central nervous system, but even lower in the hematopoietic system [89]. Since the concentration of Wnt ligands at least partially determine which Wnt target genes are transcribed, it could very well be that canonical Wnt signaling in MM does result in active transcription, but that these Wnt target genes have not yet been identified because they differ from the Wnt target genes that are activated in colon tissue. Of note, also no Wnt target genes have yet been identified in developing B cells, which also have an active canonical Wnt signaling pathway. Furthermore, MM cells are derived from the hematopoietic lineage and therefore genes which are present in epithelial tissues, including Wnt target genes, could be epigenetically silenced.

### Active Wnt signaling in multiple myeloma cells promotes drug resistance

Besides the possible effects of Wnt signaling on MM cell proliferation and subsequent MM disease progression, two studies suggest a role for Wnt in cell adhesion-mediated drug resistance (CAM-DR) of MM. Qiang et al. already reported that Wnt ligands can induce migration and invasion of MM cells by activating the non-canonical Wnt pathway mediators RhoA and PKC [83]. In 2007, Kobune et al. followed up on this study by showing that levels of Wnt3 ligand, produced in an autocrine fashion by the HMCLs, positively correlated with adhesive properties of MM cells to BM stromal cells in vitro. This finding was accompanied by stabilization of β-catenin protein levels and activation of RhoA [25]. It is generally accepted that MM cell adhesion to BM stromal cells does not only contribute to MM bone disease, but also plays a critical role in CAM-DR [119]. In accordance with this hypothesis, Kobune et al. reported that the level of Wnt3-mediated MM cell adhesion negatively correlated with the degree of drug sensitivity of MM cells to doxorubicin. Furthermore, Wnt pathway inhibition by treatment with the Wnt antagonist sFRP1, siRNA-mediated knockdown of Wnt3 ligand, or inhibition of the effector RhoA kinase ROCK by Y27632, rescued MM cell sensitivity to doxorubicin [25]. In 2011, Bjorklund et al. reported that long term exposure of HMCLs to lenalidomide increased the level of drug resistance to lenalidomide up to 2500-fold. Extensive analyses showed that this was accompanied by an up to 20-fold increase in β-catenin protein levels, a significant gain in TopFlash reporter activity and enhanced expression of the Wnt target genes cyclin-D1 and c-Myc. This was found to result from a general suppression of CK1α, one of the members of the β-catenin destruction complex, and inactivation of GSK3α/β due to increased phosphorylation of inhibitory residues. Additional treatment with recombinant Wnt3a ligand resulted in an even further increase of MM lenalidomide resistance, while shRNA-mediated knockdown of β-catenin greatly rescued drug sensitivity [120]. More recently, a follow-up study by the same group was published in which they identified the direct Wnt target gene CD44 as the downstream effector molecule of Wnt-mediated lenalidomide resistance in MM. This hyaluronan-binding protein was found to be overexpressed by lenalidomide-resistant HMCLs in vitro, and these cells showed increased adhesive potential to BM stromal cells, indicative for CAM-DR. Inhibition of CD44 reduced the adhesive properties of MM cells and rescued sensitivity to lenalidomide, reflected by an increase in apoptotic cell numbers. Interestingly, in vitro treatment of lenalidomide-resistant HMCLs with the pharmaceutical agent ATRA resulted in decreased β-catenin mRNA and protein, as well as downregulated CD44 transcription and expression. In addition, the combinatorial treatment of ATRA with lenalidomide in NOD/SCID mice that were subcutaneously engrafted with lenalidomide-resistant HMCLs, resulted in significant reduction of tumor growth and a trend towards increased survival. Also in primary PC isolates from lenalidomide-treated MM patients with relapsed or refractory disease, this combinatorial treatment of ATRA with lenalidomide showed a clear reduction in MM cell viability [121].

## The WNT pathway as a potential therapeutic target in multiple myeloma

It seems counterintuitive that MM cells require an active intrinsic canonical Wnt pathway to maintain cell survival and tumor growth, but at the same time also upregulate the canonical Wnt inhibitor Dkk1. However, Dkk1 was found to be a direct gene target of canonical Wnt signaling as well and could therefore function in a negative feedback loop, like is established for Axin2 [122]. In this perspective, Dkk1 could be acknowledged as a tumor suppressor protein. Indeed, Dkk1 expression was found to be high in early stages of active MM, but was lower or even undetectable in a subset of patients with advanced disease. This was found to be the direct result of acquired CpG island promoter methylation and resulted in increased intrinsic Wnt signaling activity in MM cells [123]. Targeting the Wnt pathway could therefore be an interesting new avenue to treat MM: (1) it could increase apoptosis of MM cells and thereby decrease tumor growth; (2) it could reduce Dkk1 levels secreted by MM cells and thereby reduce osteolytic bone disease and subsequent bone-related symptoms; (3) it could reduce MM acquired CAM-DR and thereby increase the response to established treatment regimens.

Although Wnt signaling is extensively studied and its role in malignancies is well established, biologicals targeting this cascade have not yet entered the standard-of-care procedures. This is primarily due to concerns about drug safety, as Wnt signaling is required for stem cell maintenance and tissue homeostasis during the complete course of adult life [124]. However, also the complexity of the pathway, its subtle regulation and cellular outcome makes it difficult to develop a successful strategy. Levels of β-catenin are not just under control of the Wnt-Fzd-LRP-Dvl axis, but are also regulated by alternative pathways, including HGF-Met, prostaglandin PGE2-EP2/4 and estrogen E2-ERα [125–127]. Environmental factors such as hypoxia and glucose levels were also shown to affect β-catenin activation [128, 129]. In addition, β-catenin not only facilitates the transcription of Wnt target genes by association with the LEF/TCF family members, but also co-operates with FOXO, multiple SOX transcription factors and SMADs to regulate cellular functions [130–132].

### Molecular Wnt pathway inhibitors in the clinic

Despite the aforementioned challenges, many compounds that modulate Wnt pathway activity have been identified and developed in the past decade and some have now made it to the first phases of clinical trials. The compounds LGK974 and ETC-159, also referred to as ETC-1922159, are small molecules that block the secretion of Wnt ligands by inhibition of porcupine. This membrane-bound O-acetyltransferase is required for Wnt palmitoylation, which is an essential step in ligand secretion [133]. Both drugs were initially identified by screening large libraries of small molecule compounds. Testing of LGK974 in vitro showed that this drug blocks secretion of all canonical Wnt ligands, inhibits Wnt-driven phosphorylation and activation of LRP6 and reduces Wnt target gene expression as assessed by Axin2 mRNA levels in a large panel of human head and neck cancer cell lines. Additional in vivo experiments in a Wnt-driven murine tumor model showed significant tumor reduction upon treatment with a well-tolerated dose of LGK974 [134]. Similar results were obtained for ETC-159. In vivo experiments showed that this orally-available drug reduces tumor growth in a Wnt-sensitive murine mammary cancer model, accompanied by a reduction in nuclear β-catenin levels and decreased expression of Wnt target genes, including Axin2 [135]. Both LGK974 and ETC-159 are now in phase I clinical trials for advanced Wnt sensitive solid tumors.

For the antibody-based inhibitor OPM-54F28 the first dose-escalating phase Ia clinical trial in solid tumors has been completed in 2016. OPM-54F28 consists of the cysteine-rich domain of Fzd8 fused to the IgG1 Fc region and thereby acts as a decoy receptor for extracellular Wnt ligands. Also for this compound, efficacy was validated in a Wnt-sensitive murine cancer model and additional in vivo data showed synergy in combination with the chemotherapeutic agent gemcitabine. Furthermore, a reduction in the cancer stem cell population was reported [136]. Currently, three phase Ib clinical trials are ongoing in which OPM-54F28 is combined with standard-of-care drugs for hepatocellular, ovarian and (advanced) pancreatic cancer.

Several monoclonal antibody therapies that directly target the membrane-bound receptor complex have also been developed. OMP-18R5, also referred to as vantictumab, binds to a conserved epitope of the canonical Fzd1, Fzd2, Fzd5, Fzd7, and Fzd8 receptors. Binding studies showed that this blocks the binding of Wnt3a to the extracellular domain of Fzd5, which subsequently leads to inhibition of downstream Wnt/β-catenin signaling. Treatment of human solid tumor xenografts in mice showed a clear reduction in tumor growth in a subset of the tumors and also reported significant synergistic effects in combination with standard chemotherapeutic agents, including taxol and gemcitabine [137]. Three phase Ib clinical trials are currently testing this compound in combination with standard-of-care chemotherapy in advanced solid tumors.

Most compounds developed to inhibit canonical Wnt signaling function by interfering with the downstream β-catenin-LEF/TCF complex [138]. One of the most successful agents in this category are the β-catenin-LEF/TCF antagonists PKF115-584 and the structurally-related small molecule inhibitor CGP049090. These natural compounds of fungal origin were first identified by a high-throughput ELISA-based screen. Although the exact molecular mechanism of Wnt interference has yet to be established, both compounds were found to reduce β-catenin immunoprecipitation efficiency and also inhibited the β-catenin-APC complex, suggesting a direct binding affinity for β-catenin [139]. PKF115-584 was also thoroughly characterized in a pre-clinical MM study by Sukhdeo et al. This compound was found to reduce β-catenin-LEF/TCF transcriptional activity and Wnt target gene expression, disrupts cell proliferation and induces cytotoxicity in HMCLs and primary PC isolates of MM patients. Additional in vivo data reported a decrease in tumor growth and prolonged survival in a murine xenograft model using a HMCL [84]. Although many β-catenin-LEF/TCF antagonists have proven to be successful in pre-clinical research, most of them have not yet entered clinical trials. The small molecule inhibitor PRI-724, which blocks the transcriptional co-factor CBP, is an exception. Although therapeutic specificity could be questioned, pre-clinical studies and a dose escalating phase I clinical trial in advanced/metastatic pancreatic cancer did not show major adverse events at a therapeutic dosage [140]. In 2015, a phase I/II clinical trial of PRI-724 in acute myeloid leukemia and chronic myeloid leukemia was completed. No study results have been published yet.

It is yet too early to determine which strategy of inhibiting Wnt signaling in cancer patients will be most successful and targeting the Wnt signaling pathway for the treatment of MM is still at the preclinical stage. Multiple studies have been published in the last years suggesting a range of molecules that inhibit Wnt signaling in MM, either as a single agent or in combination with currently used therapies. An example is the β-catenin inhibitor BC2059 that was shown to induce apoptosis of HMCLs, even in the presence of the protective feeder cell line HS5. Apoptosis of malignant PCs from relapsed and/or refractory MM patients induced by BC2059 was reported to be synergistically enhanced when combined with the proteasome inhibitor Bortezomib. Furthermore, NOD/SCID/gamma mice transplanted with HMCL U266 intravenously, that were treated with BC2059 showed an impaired tumor burden and an increased survival when compared to the vehicle control [141]. In addition, drugs that have been approved by the US Food and Drug Administration for the treatment of other diseases have been reported as candidates for treatment of MM. Pyrvinium pamoate (PP) is an oral anthelmintic drug that was recently reported to inhibit Wnt signaling through activation of the destruction complex member CK1α, thereby diminishing active β-catenin levels. Treatment of both the HMCL RPMI-8226 as well as primary MM cells with PP resulted in an increase in apoptosis. Combinatorial treatment with Bortezomib was also reported in this study to result in a synergistic effect on MM cell viability [142]. Although the results of these and many other studies are hopeful, challenges for moving these drugs towards treatment of MM lie ahead. As discussed previously, the Wnt pathway has an important role in keeping adult tissue homeostasis by regulating multiple stem cell populations. Long-term Wnt blockage could very well result in disruption of these processes and ultimately lead to severe side effects. In addition, resistance to Wnt inhibiting agents should also be taken into account. Whereas treatment with monoclonal antibodies can result in the occurrence of drug resistance due to downregulation of the target from the MM cell membrane, treatment of the Wnt pathway at a more downstream level can put extensive selective pressure on cells. This has indeed been described for the colorectal cancer cell line VACO6, which harbors a constitutively active Wnt pathway due to translocation of R-spondin3, resulting in a *PTPRK(e1)-RSPO3(e2)* fusion. Although clear sensitivity to the Porcupine inhibitor LGK974 was observed in vitro and in vivo in a murine xenograft model, long-term treatment led to the emergence of a resistant population. This resistant population was characterized by two novel frame-shift deletions in *AXIN1*, resulting in protein loss [143]. Although no mutations in Wnt pathway components in MM have been reported, treatment of the active Wnt signaling pathway can thus result in emergence of such mutations. This stresses the need for further research in order to determine specificity and safety of targeting the Wnt pathway in the treatment of MM.

## Conclusion

**Fig. 4:**
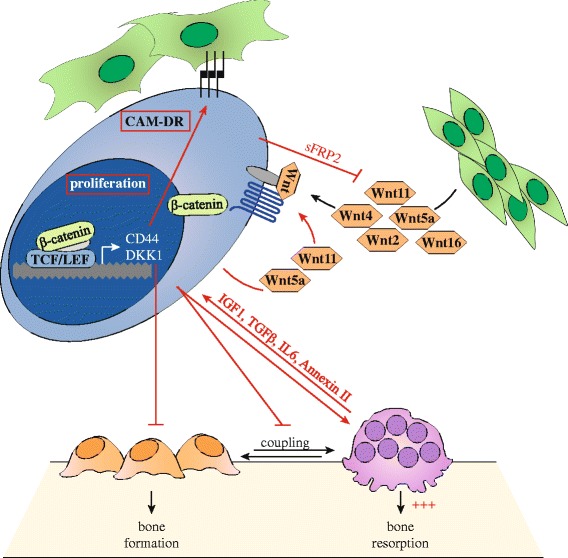
The Wnt signaling pathway is a central player in MM. MM cells have an active intrinsic Wnt pathway. This can be stimulated by Wnt ligands produced by BM cells including MSC, or in an autocrine fashion via at least Wnt5a and Wnt11. Canonical Wnt pathway activation via Fzd1, Fzd6, or Fzd7 leads to active LEF/TCF mediated transcription of Wnt target genes that promote tumor growth. The transcribed Wnt antagonist Dkk1 is secreted into the BM microenvironment and inhibits osteoblast proliferation, differentiation and survival. Combined with stimulation of osteoclast activity, this results in disruption of bone homeostasis and increased osteolytic bone resorption. The growth factors IGF-1 and TGF-β that are released from the bone matrix, together with osteoclast-secreted factors IL-6 and Annexin II, further stimulate MM cells. Expression of the Wnt target gene CD44, together with stimulation of the non-canonical Wnt pathways, stimulate adhesive properties of MM cells to BM stromal cells. This induces cell adhesion-mediated resistance of MM cells to conventional drug therapies

The Wnt signaling pathway is implicated in multiple aspects of MM disease (Fig. 4). By secreting Wnt antagonists including Dkk1, MM cells disturb the balance between bone-forming osteoblasts and bone-resorbing osteoclast. The subsequent disruption of bone homeostasis results in the development of osteolytic bone lesion. This does not only provide the MM cells with a direct and indirect growth advantage, resulting in tumor progression, but also causes MM patients to suffer from bone pain and pathologic fractures. Intrinsic canonical Wnt/β-catenin signaling has been reported to promote MM cell survival and thereby establishes disease progression. This is at least partially achieved via autocrine stimulation. In addition, both canonical and non-canonical Wnt signaling pathways are associated with acquired drug resistance. This is most likely mediated by adhesive properties of MM cells to BM stromal cells. The acquisition of MM resistance to conventional therapies is an emerging clinical problem and negatively influences patient outcome. Targeting the Wnt pathway could therefore be an interesting new avenue to treat MM. It would increase apoptosis of MM cells and as such decrease tumor growth, it would reduce secretion of Dkk1 in the BM microenvironment and thereby restrict osteolytic bone disease, and it would reduce CAM-DR in MM and thereby facilitate a more successful and prolonged response to already established drug treatment options.

## Abbreviations

BM: Bone marrow
CAM-DR: Cell adhesion-mediated drug resistance
Dvl: Disheveled
ECM: Extracellular matrix
ER: Endoplasmic reticulum
Fzd: Frizzled
HMCL: Human multiple myeloma cell line
HSC: Hematopoietic stem cell
JNK: Jun kinase
LiCl: Lithium chloride
MGUS: Monoclonal gammopathy of undetermined significance
MM: Multiple myeloma
MSC: Mesenchymal stem cells
PC: Plasma cell
PCP: Planar cell polarity
PP: Pyrvinium pamoate
sFRP: Soluble frizzled-related protein
SMM: Smoldering myeloma
WIF1: Wnt inhibitory factor 1
WRE: Wnt responsive element
